# Bridge over troubled cells: bone marrow stromal cells transfer mitochondria to boost T cells

**DOI:** 10.1038/s41392-024-02079-6

**Published:** 2024-12-13

**Authors:** Lars Fabian Prinz, Roland Tillmann Ullrich, Markus Martin Chmielewski

**Affiliations:** 1 Department I of Internal Medicine, University Hospital Cologne and Faculty of Medicine, University of Cologne, 50937 Cologne, Germany; 2 Translational Research for Infectious Diseases and Oncology (TRIO), 50931 Cologne, Germany; 3 Center for Integrated Oncology Aachen Bonn Cologne Duesseldorf, University Hospital Cologne, 50937 Cologne, Germany

**Keywords:** Cell biology, Cancer therapy, Tumour immunology, Cancer microenvironment

In an article published in *Cell* in September 2024, Baldwin and colleagues present a bone marrow stromal cell (BMSC) based mitochondrial transfer platform to combat mitochondrial dysfunction or scarcity in human T cells ex-vivo.^1^ This thorough and methodically diverse investigation results in a promising technology at a time when adoptive T cell therapies seem to run against a wall of T cell exhaustion and dysfunction in treatments targeting solid tumors.^2^

**Fig. 1:**
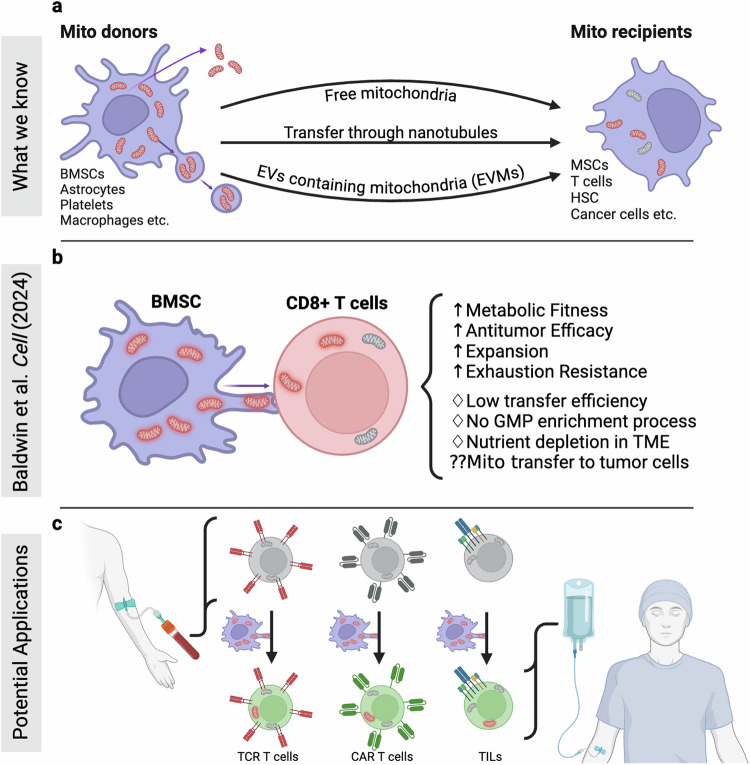
**a** Building on mitochondrial transfer techniques described in the literature, Baldwin and colleagues introduce a method to fortify CD8+ T cells with mitochondria transferred through tunneling nanotubules from bone marrow stromal cells (BMSCs). **b** The transfer results in T cells being more resistant against exhaustion and having higher anti-tumor activity in-vitro and in-vivo. **c** This could be applied to improve adoptive T cell therapies to treat patients with cancer. (Created with BioRender.com)

Intercellular mitochondrial transfer has emerged as a physiological process with therapeutic potential. It occurs naturally in a diverse combination of cell and tissue types via transient cell-cell connections, extracellular vesicles containing mitochondria (EVMs) and the capture of cell-free mitochondria (Fig. 1a). The ability of BMSCs and other cells to transfer mitochondria via tunneling nanotubes (TNTs) has been previously described, but its use to improve the metabolic fitness of CD8+ T cells is a novel and highly relevant discovery.^3^

In the study, the authors co-cultured human or mouse CD8+ T cells with species-matched BMSCs and observed the formation of TNTs connecting both cell types (Fig. 1b). Using scanning electron microscopy and confocal imaging, they confirmed that mitochondria labeled with a dsRed fluorescent marker in BMSCs were transferred to CD8+ T cells via established nanotubes. Notably, the transferred mitochondria were functional, as evidenced by intact membrane potential.

The acquisition of BMSC mitochondria led to a significant increase in the mitochondrial mass and respiratory capacity of CD8+ T cells. Specifically, mitochondrial transfer enhanced basal respiration and spare respiratory capacity, indicating improved metabolic capacity. When the researchers impaired mitochondrial function in BMSCs using ethidium bromide, the benefits to T cells were abolished, ruling out that fitter T cells simply being more accepting of mitochondrial transfer is the deciding factor.

To assess the therapeutic potential of mitochondrial transfer, the researchers used a B16_KVB_ allograft mouse model of melanoma cells that could be targeted by CD8+ T cells expressing a transgenic T cell receptor (TCR). CD8+ T cells that had acquired BMSC mitochondria (Mito+ cells) were more effective in reducing tumor growth and prolonging survival compared to T cells without transferred mitochondria (Mito− cells). Mito+ T cells showed enhanced proliferation, increased infiltration into tumor tissues, and reduced apoptosis within the tumor microenvironment.

Single-cell RNA sequencing and flow cytometry analyses revealed that these Mito+ T cells resisted terminal exhaustion and maintained a more functional phenotype, with lower expression of immune checkpoint receptors such as PD-1, LAG3, and TIGIT. They also exhibited higher levels of effector molecules like granzyme B, indicating a robust cytotoxic capacity.

Extending their findings to human cells, Baldwin et al. demonstrate that mitochondrial transfer improved the function of CD19-specific chimeric antigen receptor (CAR) T cells and tumor-infiltrating lymphocytes (TILs). In vitro, Mito+ CAR T cells exhibited enhanced cytotoxicity towards leukemia cells and maintained this activity over multiple rounds of stimulation, suggesting resistance to exhaustion. Furthermore, the authors demonstrate in an acute lymphoblastic leukemia NXG mouse model strongly enhanced cytotoxic capacity of Mito+ CAR T cells against leukemic cells in comparison to Mito- CAR T cells or aCD19-CAR CD8 cells. Most strikingly, treatment with Mito+ CAR T cells improved overall survival of NXG mice bearing acute lymphoblastic leukemia in comparison to Mito- CAR T cells or monocultured aCD19-CAR CD8 cells.

The authors also present Talin 2 (TLN2) as a key regulator of TNT-mediated mitochondrial transfer, as it was one of the few genes upregulated in both human and murine Mito+ cells. Accordingly, the authors demonstrate that targeted CRISPR-Cas9-based TLN2 knockout led to disruption of mitochondrial transfer.

Low transfer efficiencies of 12.6% (6.2–24.7%) present a challenge for clinical use, as pointed out in the paper. Thus, the authors suggest that transfer efficiencies have to be improved through process optimizations. Alternatively, identifying surrogate surface markers for mitochondrial content would enable post-transfer FACS enrichment. However, even without significant progress in these technical areas, the fact that small subpopulations of a CAR T product can be critical to response^4^ suggests that the enrichment performed for significant in-vitro and in-vivo results might not be necessary when treating patients.

There are also more profound challenges for translation into clinical practice: First, the exhaustion of T cells in the tumor microenvironment (TME) is partly due to substrate depletion. Even though Mito+ cells demonstrated higher total metabolic capacity than Mito- cells in B16_KVP_ tumor bearing mice, different tumors with higher mitotic and metabolic activity, lower vascularization or a less permissive tumor stroma might still outcompete Mito+ T cells for nutrients. Second, intercellular mitochondrial transfer from T cells to tumor cells has been described^5^ and although the authors note that this is not observed in their experimental system, it might be a relevant and possibly prohibitive factor for clinical applications. Third, even though mitochondria do not cause alloreactivity, BMSCs would likely need to be separated from an adoptive T cell product after transfer while still allowing cell-cell contact during, since free-floating mitochondria from BMSCs did not transfer.

If these factors can be successfully addressed, mitochondrial transfer to CD8+ T cells could be a key technology to improve current adoptive T cell therapies and expand their scope (Fig. 1c). TIL therapy in particular stands to benefit from exhaustion-rescue or protection, since cells are extracted from the TME and have already been exposed to tumor-intrinsic suppression mechanisms. This could unleash their potential as polyclonal T cells that are not limited to one or two target epitopes.

Less obviously, this discovery could also enable non-cell-based treatments that modulate physiological mitochondrial transfer in patients. L-778,123, similar substances, or TLN2 regulators could be used systemically with the intent of abrogating mitochondrial transfer to malignant cells or to increase transfer to effector cells in-vivo. Pretreatment of adoptive T cell products is also a viable avenue of investigation.

In conclusion, Baldwin et al. have devised a potent platform to enhance adoptive T cell metabolic fitness and improve their function in in-vitro and in-vivo models. Challenges to translation exist, and the clinical need for exhaustion-resistant adoptive T cell therapies urge the importance of investigating and removing these roadblocks.
